# Preoperative Embolization of a Solitary Fibrous Tumor Originating from External Auditory Meatus: A Case Report with Literature Review

**DOI:** 10.3390/diagnostics11010062

**Published:** 2021-01-02

**Authors:** Urszula Maria Ciochon, Grethe Schmidt, Ruben Juhl Jensen, Anand C. Loya, Lars Birger Lönn, Nitesh Shekhrajka

**Affiliations:** 1 Department of Diagnostic Radiology, Copenhagen University Hospital (Rigshospitalet), 2100 Copenhagen, Denmark; ruben.juhl.jensen.02@regionh.dk (R.J.J.); lars.birger.loenn@regionh.dk (L.B.L.); nitesh.shekhrajka@regionh.dk (N.S.); 2 Department of Plastic Surgery and Burns Treatment, Copenhagen University Hospital (Rigshospitalet), 2100 Copenhagen, Denmark; grethe.schmidt@regionh.dk; 3 Department of Pathology, Copenhagen University Hospital (Rigshospitalet), 2100 Copenhagen, Denmark; anand.chainsukh.loya@regionh.dk

**Keywords:** solitary fibrous tumor, endovascular embolization, preoperative embolization, retro-auricular tumor, microspheres

## Abstract

Solitary fibrous tumors (SFTs) are mesenchymal, fibroblastic tumors with mostly favorable, but still unpredictable prognosis. Their rarity and occurrence at a variety of locations coupled with variable histological appearance make the diagnosis a challenge. This can be resolved by histological and immunohistochemical analysis on the histologic material eventually coupled with demonstration of *NAB2-STAT6* gene fusion by next generation sequencing (NGS) analysis. Tumor removal with clear surgical margins is sufficient for complete cure in most cases. Percutaneous transcatheter embolization in well-vascularized lesions may minimize the risk of bleeding during subsequent removal. In this article we present a rare case of SFT arising from the external auditory canal and treated with preoperative endovascular arterial embolization. A literature review with focus on diagnostics and treatment of this entity in the head and neck region is following.

## 1. Introduction

Solitary fibrous tumors (SFT) are rare, slow-growing type tumors that can arise in all body regions and that comprise less than 2% of all soft tissue masses [1]. The most common location is the pleura, while 6–12% of SFTs arise from the head and neck region [2,3], with oral cavity [4], sinonasal tract and orbit [2,5] described as the most common sites in this region. SFT in the external auditory canal is an exceptionally rare occurrence with only few described cases [6,7,8,9]. SFTs can appear well-vascularized and depending on the vascular supply, a preoperative endovascular arterial embolization can be performed to simplify subsequent tumor removal with clean margins. In this article we present a SFT originating from external auditory canal treated with percutaneous transcatheter embolization technique in combination with surgery. Only one similar case has previously been described [9].

## 2. Case Report

A 28-year-old man known with alopecia areata was referred for ear-nose-throat assessment due to a six month history of a slow-growing palpable, painless and pulsatile mass behind the right ear, protruding into the external auditory meatus, causing a pulsatile tinnitus and a decreased hearing. Physical examination revealed a visible, firm and mobile mass in concha of the right auricle without any changes in the overlying skin. Audiometry showed no abnormalities and the patient had no lightheadedness. No signs of cranial nerve involvement were noted.

**Figure 1:**
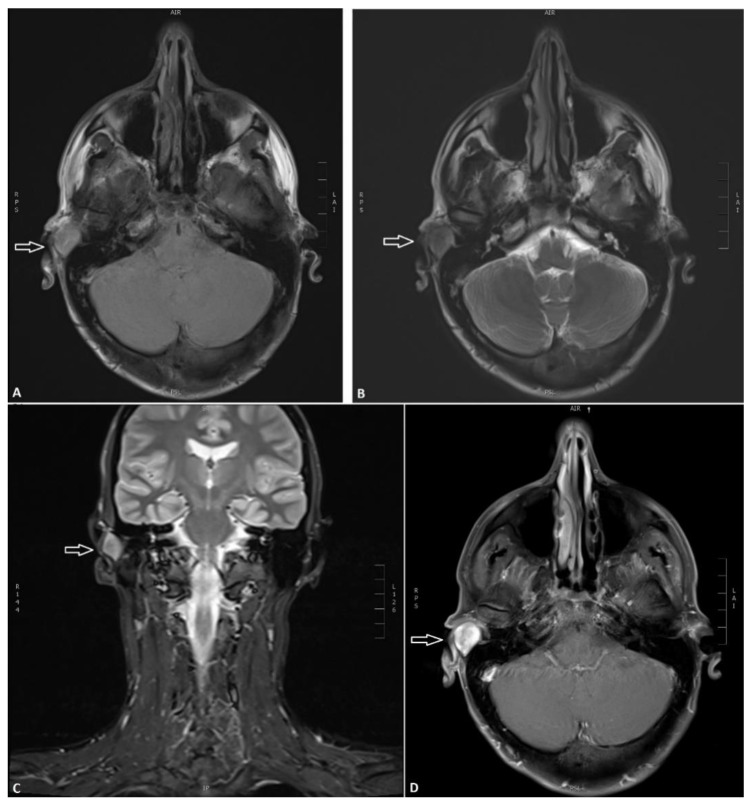
Initial MR scanning. (**A**) Axial T1-weighted MR showing a slightly hyperintense tumor with a retroauricular location on the right side (arrow). (**B**) Axial T2-weighted MR showing inhomogeneous signal corresponding to the tumor. (**C**) Coronal short tau inversion recovery (STIR) with the slightly hyperintense tumor. (**D**) Axial contrast-enhanced T1-weighted MR showing a strong contrast enhancement of the tumor with some central non-enhancing sites.

A 3 Tesla magnetic resonance (MR) scanning (Siemens, Frankfurt am Main, Germany) before and after intravenous (IV) contrast administration revealed a well-defined retroauricular tumor of 2 cm in diameter causing mass effect on the auricula and a near-total occlusion of the external auditory meatus (Figure 1A–D). It had an inhomogeneous signal on T2-weighted images, slightly hyperintense on T1-weighted series and on STIR (short tau inversion recovery) images, and contained a central 4 mm area with restricted diffusion. The tumor had a strong enhancement after IV contrast administration except a few small central non-enhancing sites. No large vascular structures or flow voids in relation to the tumor and no signal changes in the underlying temporal bone including the middle ear and mastoid air cells were seen. The inner ear, parotid glands as well as other salivary glands were normal. No suspicious changes in the nasal and oral cavity, in the pharynx or in the thyroid gland and no pathological or enlarged lymph nodes in the head and neck region were encountered. Other facial regions, brain, cranial bones as well as the cervical spine and cervical medulla were also without any abnormality.

**Figure 2:**
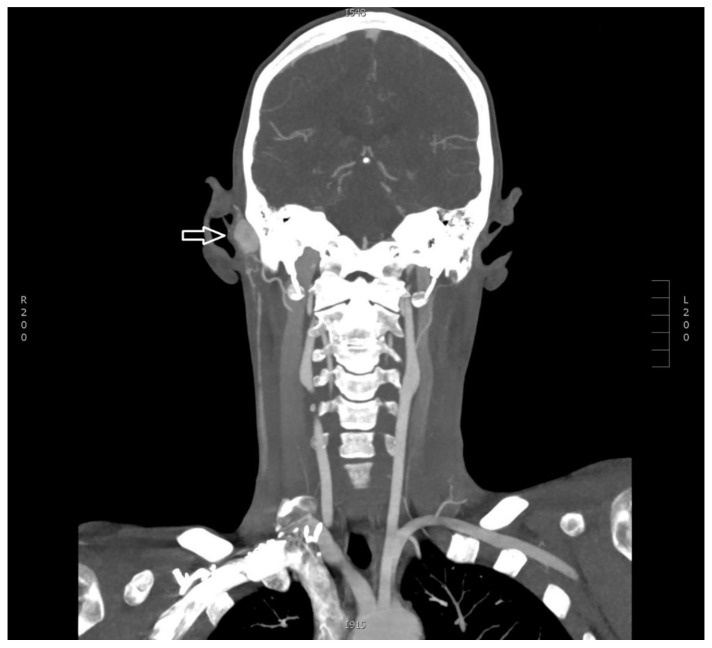
Coronal maximum intensity projection (MIP) with multiplanar reconstruction (MPR) showing the right-sided contrast-enhancing tumor (arrow) supplied by arterial branches from the right external carotid artery and with surrounding veins draining into the right external jugular vein.

**Figure 3:**
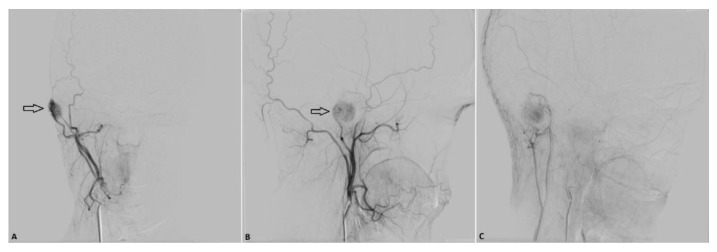
Diagnostic digital subtraction angiography (DSA). (**A**,**B**) Anteroposterior (AP) (**A**) and lateral projection (**B**) in the arterial phase after injection in the right external carotid artery showing a well-vascularized tumor (arrow) with arterial supply from the posterior auricular artery on the right side. (**C**) Lateral projection in the venous phase showing venous drainage from the tumor into the external jugular vein on the right side.

Based on the described tumor morphology, the patient’s symptoms and desire to have the mass removed, a supplementary computed tomography (CT) angiography of the head and neck was performed as a part of the preoperative preparations. The scanning revealed a very quick contrast enhancement of the tumor during the early arterial phase (Figure 2). Several small arterial branches from the external carotid artery supplied the tumor with some dilated extracranial veins around the lesion, presumed to be the venous drainage. The anterior and posterior intracranial circulation was normal. No osseous changes were seen around the tumor. A supplementary digital subtraction angiography (DSA) was ordered based on suspicion of an arteriovenous malformation (AVM) or a venous varix and showed a tumor blush corresponding to the external auditory canal, with arterial supply originating from the right posterior auricular artery and the right ascending pharyngeal artery, draining into the right external jugular vein (Figure 3A–C). No dural arteriovenous fistula or aneurysm was seen. Because of the rich vascularity, a preoperative endovascular embolization of the lesion was scheduled to facilitate the surgery.

**Figure 4:**
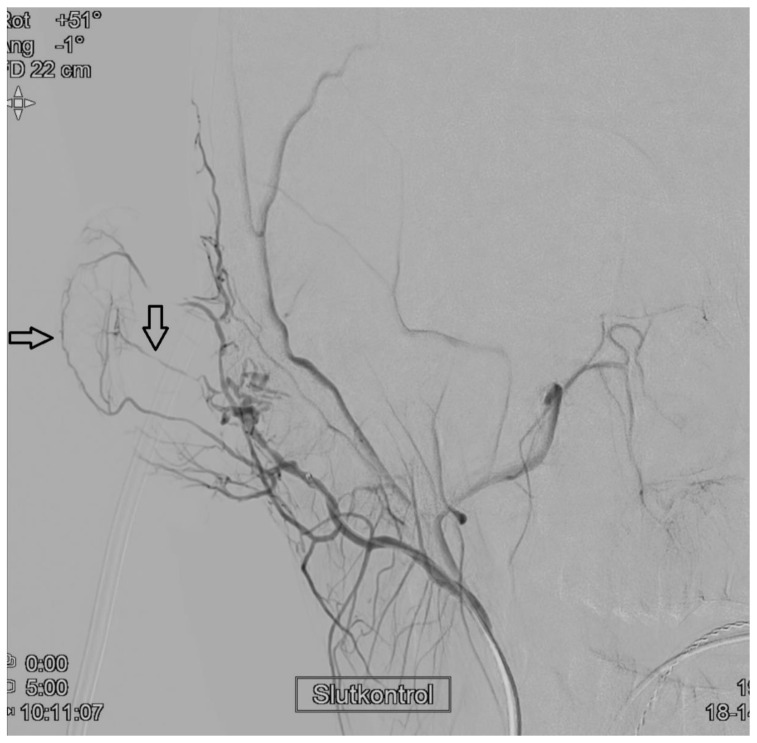
Postembolization angiography. A control DSA with injection in the right external carotid artery (AP-projection) showing a good angiographic result of the tumor embolization, with contrast filling of arterial branches to the external ear (arrows).

After puncture in the right common femoral artery (CFA) a 4 F sheath was placed with subsequent catheterization of the right external carotid artery. Selective angiography confirmed tumor blush of the lesion. The main supplying arterial branch to the tumor originated at a sharp angle and the 2.4 French microcatheter was downsized to a 1.9 French. A 0.04 inch guidewire was introduced into the main feeding artery, but the catheter could only follow the wire into the periphery of the tumor blush. Due to the risk of external ear necrosis, only a small portion of the particles (1,2 mL Embozene, Varian, Palo Alto, CA, USA, 250 microns) was injected into the intratumoral vessels. Postembolization angiography showed a good result with complete regression of the tumor blush with contrast filling of feeding arteries to the external ear. A minute non-target embolization to the auricle could not be completely ruled out (Figure 4), but no skin erythema was noted. Hemostasis of CFA was achieved by manual compression.

**Figure 5:**
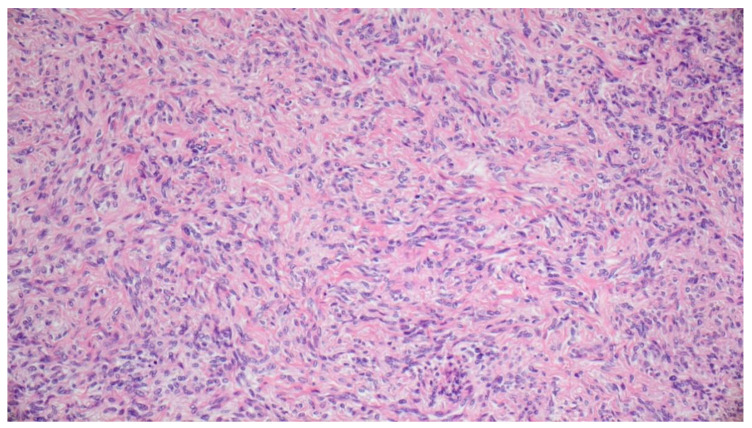
Photomicrograph showing a spindle cell tumor. Cells are arranged in a “patternless pattern”. Note the abundant stromal collagen including “ropy collagen”. There is no atypia or mitosis (hematoxylin and eosin stain, 20×).

**Figure 6:**
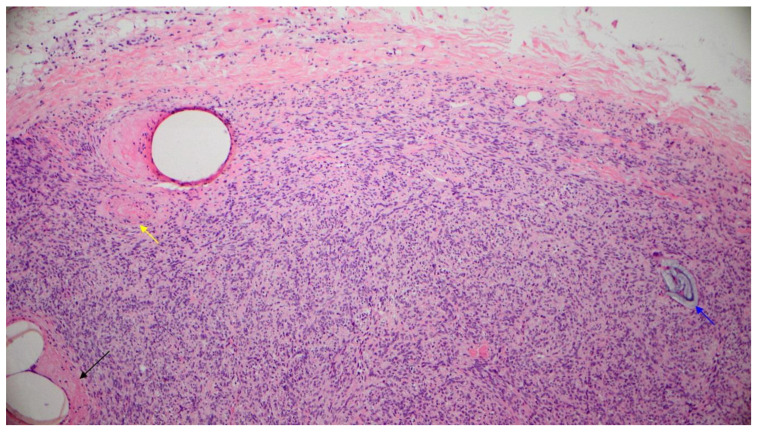
Encapsulated tumor with thrombosed vessels (black and yellow arrows). There is also presence of embolizing material used (blue arrow) (hematoxylin and eosin stain, 10×).

**Figure 7:**
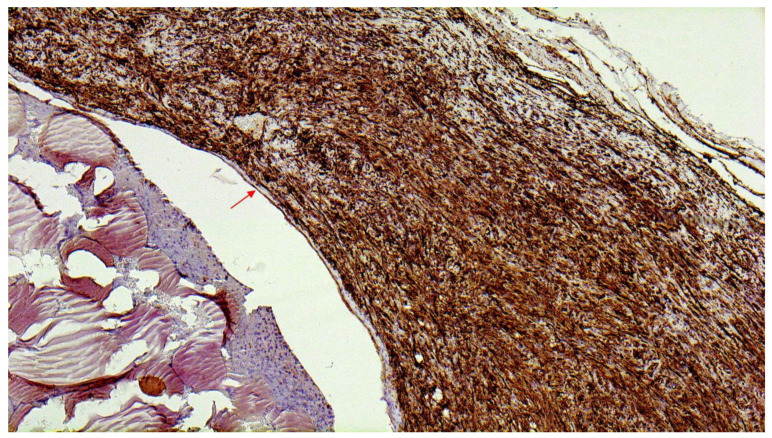
Tumor cells with strong diffuse positive reaction for CD34. Note an embolized vessel lined by CD34 positive endothelial cells (red arrow) as internal control (immunohistochemical stain, 10×).

**Figure 8:**
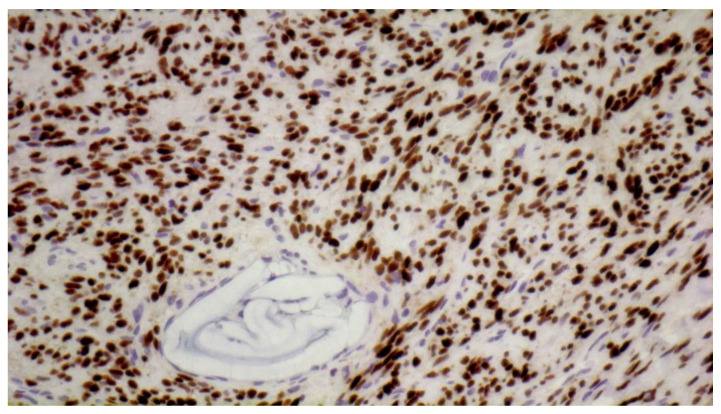
Strongly positive nuclear STAT6 immunohistochemical stain with adjacent vessel containing embolizing material (immunohistochemical stain, 20×).

On the following day, tumor removal was performed by the plastic surgeon. The tumor was encapsulated and removed in total through a retroauricular incision. Histology showed tumor cells with oval, mild to moderate pleomorphic nuclei and moderate to sparse amount of eosinophilic cytoplasm. The tumor cells were arranged haphazardly in a “patternless pattern” separated by “ropy collagen” and vessels (Figure 5). Many vessels were seen filled with the embolic material and many capillaries contained thrombi and fibrin (Figure 6). The number of mitoses was about 2 per 10 high power fields and proliferation rate with Ki-67 was 3%. Subsequent immunohistochemistry showed tumor cells strongly positive for CD34 (Figure 7) and STAT6 (Figure 8). There was negative reaction for actin, desmin, S-100 and SOX10. Thus a diagnosis of solitary fibrous tumor was rendered.

**Figure 9:**
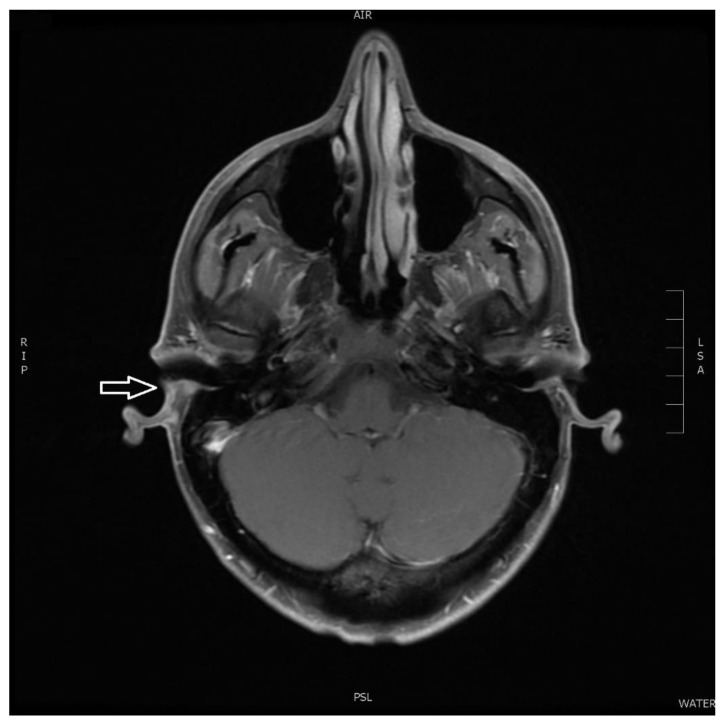
Follow-up MR performed 9 months after tumor removal. Axial postcontrast T1-weighted sequence showing sparse scar tissue in the posterior wall of the right external auditory meatus (arrow) and no signs of tumor recurrence.

A MR performed nine months after tumor removal confirmed no tumor recurrence (Figure 9). Due to the unpredictable recurrence risk of SFTs the patient will be followed for the next five years.

## 3. Discussion

Previously classified as hemangiopericytoma or benign or solitary mesothelioma, SFT is a mesenchymal tumor largely with unpredictable behavior. Most often it affects individuals between 30 and 40 years of age and no sex predominance has been observed [6]. The incidence is increasing, most likely due to high-end diagnostic modalities and increased awareness coupled with routine follow-up imaging for other medical ailments [6]. The tumor is asymptomatic until it compresses nearby structures. Generally it appears well-differentiated from surrounding structures on MR or CT. Erosion or remodeling of the underlying bone can happen but does not necessarily indicate malignancy. On CT scan the tumor possesses various densities depending on the amount of collagen and myxoid tissue content [10,11]. On precontrast T1-weighted MR it tends to be iso- or hypointense to the musculature and brain, and on precontrast T2-weighted images it is homogenously hyperintense or sometimes heterogeneous depending on the cellularity, the amount of collagen and the presence of degeneration or hemorrhage [11]. Due to rich vascularity, the tumor has an avid, strong homogenous enhancement after IV contrast administration both on CT and MR. Heterogeneous contrast enhancement can also be encountered, and in case of pleural SFTs is typically seen in the malignant changes and in 60% of benign tumors [12]. The pattern of contrast enhancement depends on the tumor cellularity, the amount of connective tissue elements as well as the presence of degenerative changes. In addition, SFTs tend to present with non-restricted diffusion on DWI and show a rapid enhancement with slow washout pattern on dynamic contrast-enhanced (DCE)-MR [13].

Histopathologic analysis of SFTs shows randomly oriented, alternating hypo- and hypercellular areas consisting of spindle-shaped to ovoid cells together with collagenous or sometimes myxoid stroma and a variable amount of dilated, “staghorn”-like, thin-walled vascular structures [6,8]. Histological variants include cellular, myxoid, fat-forming, dedifferentiated, malignant and giant-cell SFT [14]. The *NAB2-STAT6* fusion gene is definitional for SFTs and can be represented by STAT6 immunohistochemical stain, which is very reliable and thus diagnostic of SFT [15]. CD34 is a characteristically overexpressed antigen in SFTs [16], though it can be lost in some malignant SFTs [17]. Significant number of cases show also a CD99 and B-cell lymphoma protein 2 (Bcl-2) positivity, but are usually negative for S-100, desmin, nuclear β-catenins, cytokeratins and other muscular, vascular, neural and epithelial markers [8,9,13,17,18]. Immunohistochemical analysis distinguishes these tumors from other microscopically similar spindle-cell and mesenchymal neoplasms that also can be found in the external auditory meatus, such as schwannoma or fibroma [6,16]. Benign fibrous histiocytoma, sarcomatoid carcinoma as well as well-vascularized pathologies like vascular malformation, vascular metastasis, angiosarcoma, high-grade sarcoma or venous varix also have to be excluded [18,19]. The Ki-67 marker can be used to assess the proliferation rate [20].

Local recurrence after incomplete resection, malignant dedifferentiation and metastases are possible in 10% of SFTs in all locations, even many years after tumor removal and independently of its malignant or benign characteristics [17]. As a consequence, the 2013 World Health Organization classification of soft tissue tumors ranges SFTs as tumors with intermediate biological potential (rarely metastasizing) [21]. A slightly worse prognosis with higher frequency of recurrence is reported after removal of extrathoracic SFTs [6,17], especially if associated with the *NAB2ex6-STAT6ex16/17* fusion variant [22]. Mild nuclear atypia and few mitoses can occur in benign lesions and intratumoral inflammation is frequent, but does not necessarily indicate malignancy [6]. Adverse prognostic factors in head and neck SFTs include patient age ≥55 years, tumor size >5 cm, deep tumor location, nuclear pleomorphism, high nuclear-to-cytoplasma ratio, high mitotic counts (>4 per 10 high-power fields), high cellularity, intratumoral hemorrhage, necrosis, infiltrative growth, positive surgical margins, disseminated disease and non-surgical treatment [3,6,17,23,24,25]. However, even if the tumor possesses adverse prognostic features, it will not necessarily behave as a malignant tumor. The same concerns tumors with benign characteristics—their potentially aggressive course cannot be fully excluded. Positive surgical margins seem to predict the recurrence more than unfavorable microscopic appearance of the tumor [16]. Moreover, it was observed that extrathoracic SFTs recur more often than thoracic SFTs regardless of the presence of adverse features mentioned above [17].

Complete surgical removal is the treatment of choice as the tumor can exhibit an invasive course which is difficult to predict [6]. Complete removal alone is usually enough for permanent cure, even if the tumor shows malignant features [16]. However, total removal can sometimes be challenging in the head and neck region due to the complex anatomical conditions and surrounding vital structures. A longer course with follow-up imaging is advised even in completely removed tumors with benign characteristics owing to SFTs unpredictable prognosis with a potential for recurrence and metastasizing.

Depending on the tumor vascularity, surgical removal can be supplemented with endovascular embolization to reduce intraoperative bleeding [6,9,26], thus enabling a better visualization of anatomical relations to ensure clear surgical margins. Additionally, the resulting necrotic softening of the tumor can simplify the resection [26]. The use of spherical particles, polyvinyl alcohol (PVA) and Onyx (ethylene vinyl alcohol copolymer dissolved in dimethyl sulfoxide and micronized tantalum powder; EV3, Irvine-CA) has been described in transarterial embolization of the head and neck SFTs [20,27]. Just like PVA, microspheres (including Embozene that was used in our case) block the vessel inducing inflammatory reaction, angionecrosis and fibrosis. Contrary to PVA, microspheres are symmetrical, very precisely sized and non-aggregating particles. Particle reflux and nontarget embolization can happen with the use of both agents [28]. The cohesive and nonadhesive properties of Onyx with relatively slow precipitation allow a good embolization control. Additionally, the same microcatheter can be used both for Onyx injections and repeated angiograms with reduced risk of blocking the microcatheter and glueing it into the vessel, as is the case with adhesives. This allows deeper Onyx penetration into the vascular bed within the tumor with higher embolization rates, lowers the risk of nontarget embolization and allows longer procedure times [20,28]. However, an important disadvantage of this agent is the occurence of potentially dangerous sparking and prolonged combustion with the use of monopolar electrocautery during cutting the Onyx-filled vascular bed [29]. A similar phenomenon was also described in vitro using bipolar electrocautery with higher energy settings. Micronized tantalum powder was reported as the the source for ignitability of Onyx [29].

Alternative treatment possibility is percutaneous tumor cryoablation. Its choice depends on the tumor location allowing percutaneous access. In the head and neck region, the technique has been described in the treatment of a buccal space SFT [4], but not in the treatment of auricular SFTs. The advantages of this technique include minimal or absent surgical scar, pain-alleviating effect of freezing, short recovery time and the possibility of real-time visualization of the tumor with ultrasound, CT or MR during and at the end of the procedure, allowing monitoring of the tumor margins, treatment success and avoiding unintentional harm to neighboring structures. Large or irregularly shaped tumors can be treated with additional cryoablation needles during the same procedure [4].

The surgical treatment can be supplied with radiotherapy if the SFT was not removed completely or if the tumor shows malignant features [30]. SFTs respond poorly to chemotherapy [17], thus early tumor removal with clear surgical margins should be the ultimate goal.

## 4. Conclusions

Solitary fibrous tumor is a rare tumor that can arise in all body regions. It has a similar radiomorphology to many other space-occupying lesions of the external auditory canal and the definitive diagnosis is made by histologic and immunohistochemical analysis. Early tumor removal with clear surgical margins is important as the tumor has unpredictable prognosis regardless of its macro- and microscopic appearance. A longer follow-up is therefore advised. In well-vascularized tumors an endovascular embolization can aid in subsequent tumor removal.

## Data Availability

Data sharing not applicable.
